# Mechanochemical Coupling of Catalysis and Motion in a Cellulose-Degrading Multienzyme Nanomachine

**DOI:** 10.1021/acscatal.3c05653

**Published:** 2024-02-06

**Authors:** Krisztina Zajki-Zechmeister, Manuel Eibinger, Gaurav Singh Kaira, Bernd Nidetzky

**Affiliations:** † Institute of Biotechnology and Biochemical Engineering, Graz University of Technology, Petersgasse 10-12/1, Graz 8010, Austria; ‡ Austrian Centre of Industrial Biotechnology, Petersgasse 14, Graz 8010, Austria

**Keywords:** polysaccharide materials, cellulosomes, nanomachines, macromolecular motion, mechanochemical coupling

## Abstract

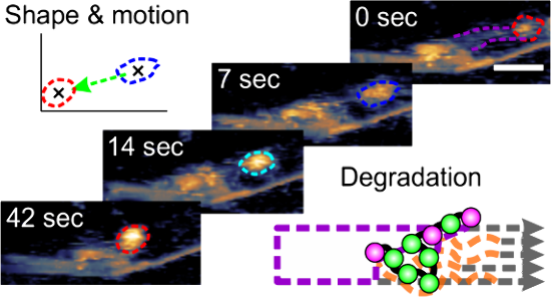

The cellulosome is a megadalton-size protein complex that functions as a biological nanomachine of cellulosic fiber degradation. We show that the cellulosome behaves as a Brownian ratchet that rectifies protein motions on the cellulose surface into a propulsion mechanism by coupling to the hydrolysis of cellulose chains. Movement on cellulose fibrils is unidirectional and results from “macromolecular crawl” composed of dynamic switches between elongated and compact spatial arrangements of enzyme subunits. Deletion of the main exocellulase Cel48S eliminates conformational bias for aligning the subunits to the long fibril axis, which we reveal as crucial for optimum coupling between directional movement and substrate degradation. Implications of the cellulosome acting as a mechanochemical motor suggest a distinct mechanism of enzymatic machinery in the deconstruction of cellulose assemblies.

## Introduction

**Figure 1:**
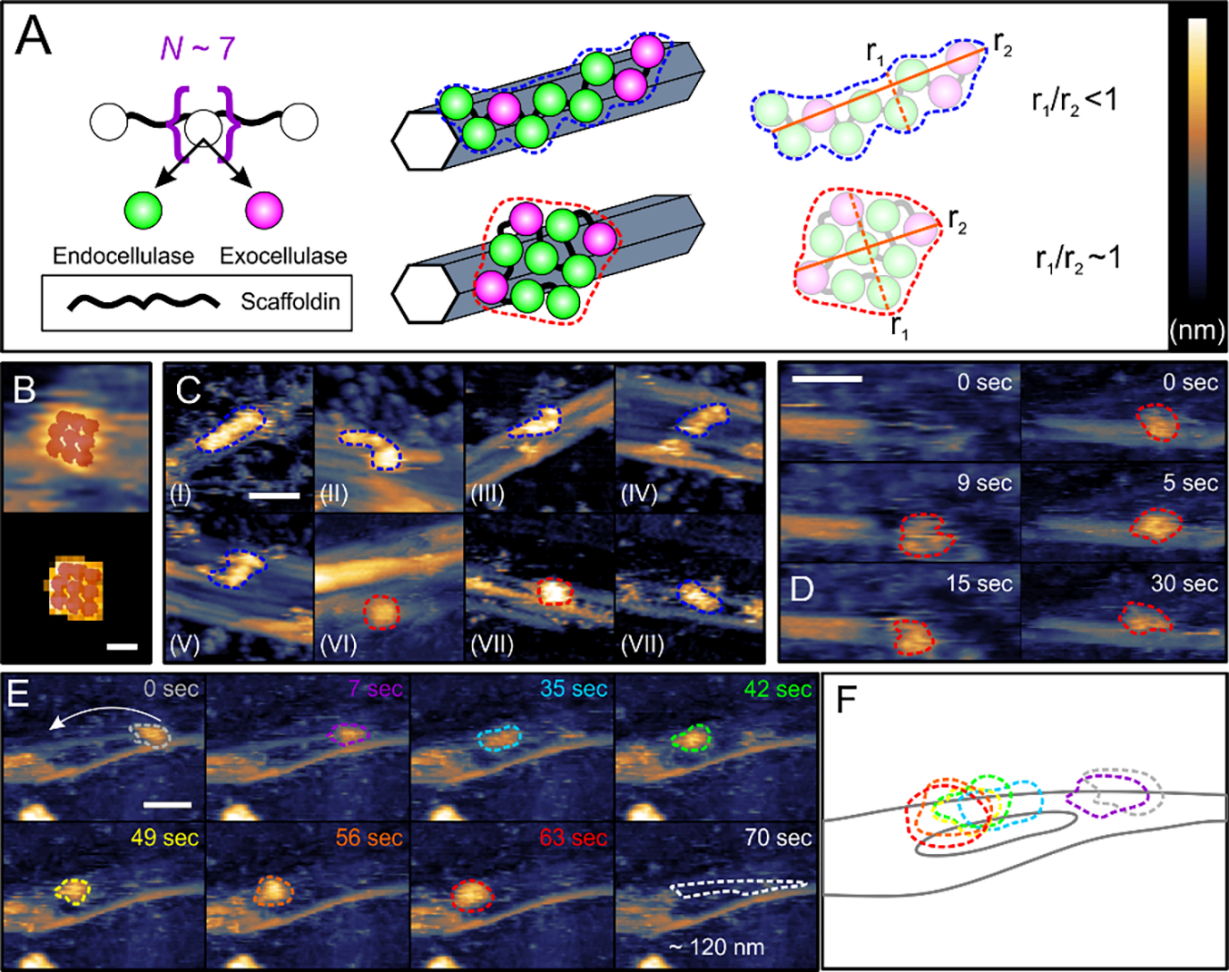
Cellulosome multienzyme complex analyzed on the cellulose surface. (A) Schematic of the *C. thermocellum* cellulosome quaternary organization with nine endo- and exocellulase subunits assembled randomly on the scaffold protein. The ratio of orthonormal vectors *r*_1_ and *r*_2_ is used as a descriptor of the cellulosome shape. (B) Surface area occupied by a round-shaped cellulosome on cellulose, as revealed by AFM in a liquid environment. The experimental height image (upper panel) is superimposed with a simulated AFM image (lower panel) of a hypothetical cellulosome composed of nine tightly arranged subunits of the exocellulase Cel48S (PDB code 5yj6). (C) Different cellulosome shapes from elongated (framed blue) to round (framed red). (D) Single cellulosome (framed red) sitting in place (center of mass change <20 nm), while its shape fluctuates. (E, F) Single cellulosome (framed in color) moving directionally on a cellulose fibril that is degraded in the process. Different colors identify the position and shape of the cellulosome in panel (F). The degraded fibril is indicated in the white (E) or gray frame (F). Scale bars are 5 nm (B) and 50 nm (C–E). The false color scale used throughout is in panel (A). Height ranges are 15 nm (I and II), 10 nm (III and IV), 11 nm (V), 25 nm (VI), and 8 nm (VII and VIII) in panel (C) and 9/14 nm for panels (D)/(E), respectively.

Digestion of insoluble carbohydrate fiber by the gut microbiota relies on the catalytic work of cell-associated protein complexes known as “cellulosomes”.^1,2^ The cellulosomes are megadalton-size modular assemblies of multiple subunits of polysaccharide-hydrolyzing enzymes that are spatially organized on a noncatalytic scaffold protein (Figure 1A).^3,4^ The enzymatic subunits constitute a core set of activities required for the complete digestion of the fiber polysaccharides, mainly cellulose and hemicellulose.^5−9^ The different enzymes synergize in attacking the substrate structures present in the fiber material under degradation.^8,10−12^ Enzyme colocalization in the protein complex is thought to benefit the biochemical synergy through proximity effects.^13−15^ The cellulosome represents the natural paradigm of a multienzyme nanomachine for efficient degradation of insoluble polysaccharides.^1,2,16,17^ By extension, it inspires synthetic biology to engineer scaffold-supported enzyme assemblies for the deconstruction of natural and synthetic materials.^18−20^

Molecular machines operate under the input of energy to drive equilibrium fluctuations of the configurational state into slower out-of-equilibrium functional transitions.^21−23^ The directional movement of processive glycoside hydrolases on their fibrillar-crystalline polysaccharide substrates exemplifies such a kind of transition, based on a catalysis-driven transduction of chemical into mechanical energy.^24−30^ The moving single enzyme behaves as an autonomous molecular motor that progresses around its chemomechanical cycle by the polysaccharide chain depolymerization catalyzed. Kinetic asymmetry required to generate preference to move directionally^21,23^ appears to result from the lack of reversibility of the steps of hydrolytic cleavage and soluble product release. However, how can the idea of a kinetically gated molecular motor function in polysaccharide degradation be applied to a large assembly of multiple enzymes? Is coupling between catalysis and motion on the fibril surface mechanistically important to an efficient deconstruction of the substrate material? Here, we analyze the dynamic behavior of the cellulosome on the cellulose surface and show its relationship with cellulose degradation.

## Results

### Conformational States of Cellulosomes on the Cellulose Surface

We used high-speed (up to 2 frames/s) real-time atomic force microscopy (AFM) in a liquid environment to observe the interaction of individual cellulosomes with isolated fibers of bacterial crystalline cellulose in their most native, never-dried state (see the SI: Atomic Force Microscopy, Preparation, and Isolation of Bacterial Cellulose Fibers). We tracked the position and shape of single cellulosomes (*N* = 512) on cellulose and monitored the local degradation of the fiber nanostructure by each of them (see the SI: Cellulosome Analysis Software). The cellulosomes used are from *Clostridium thermocellum*,^12,31^ and in the predominant form observed, they comprise nine enzymatic subunits of the fully occupied scaffold protein^16^ (Figure 1A,B and see the SI: Preparation of Cellulosomes and In Silico Size Estimation of Cellulosomes).

The cellulosomes adopt a wide variety of quaternary conformations on cellulose due to the flexible spatial arrangement of their subunits in contact with the solid surface. Based on its enveloping shape, the conformation can lie anywhere between extremes of completely elongated and completely round (Figure 1A,C,D). The individual cellulosomes show dynamic changes in their conformation. Frequently such changes coincide with, and appear to be connected to, a directional change of the cellulosome’s position on the fiber, covering distances of up to 120 nm (Figure 1E,F). The substrate is often degraded massively in the process. Random fluctuations of the internal structural organization of the cellulosome cannot promote directional movement.^23^ We therefore hypothesized coordinated functional coupling between catalysis, conformational dynamics, and directional motion of the cellulosome nanomachine, with possible importance for efficient deconstruction of the cellulose fiber.

### Correlation between Cellulosome Dynamics and the Cellulose Surface Nanostructure

**Figure 2:**
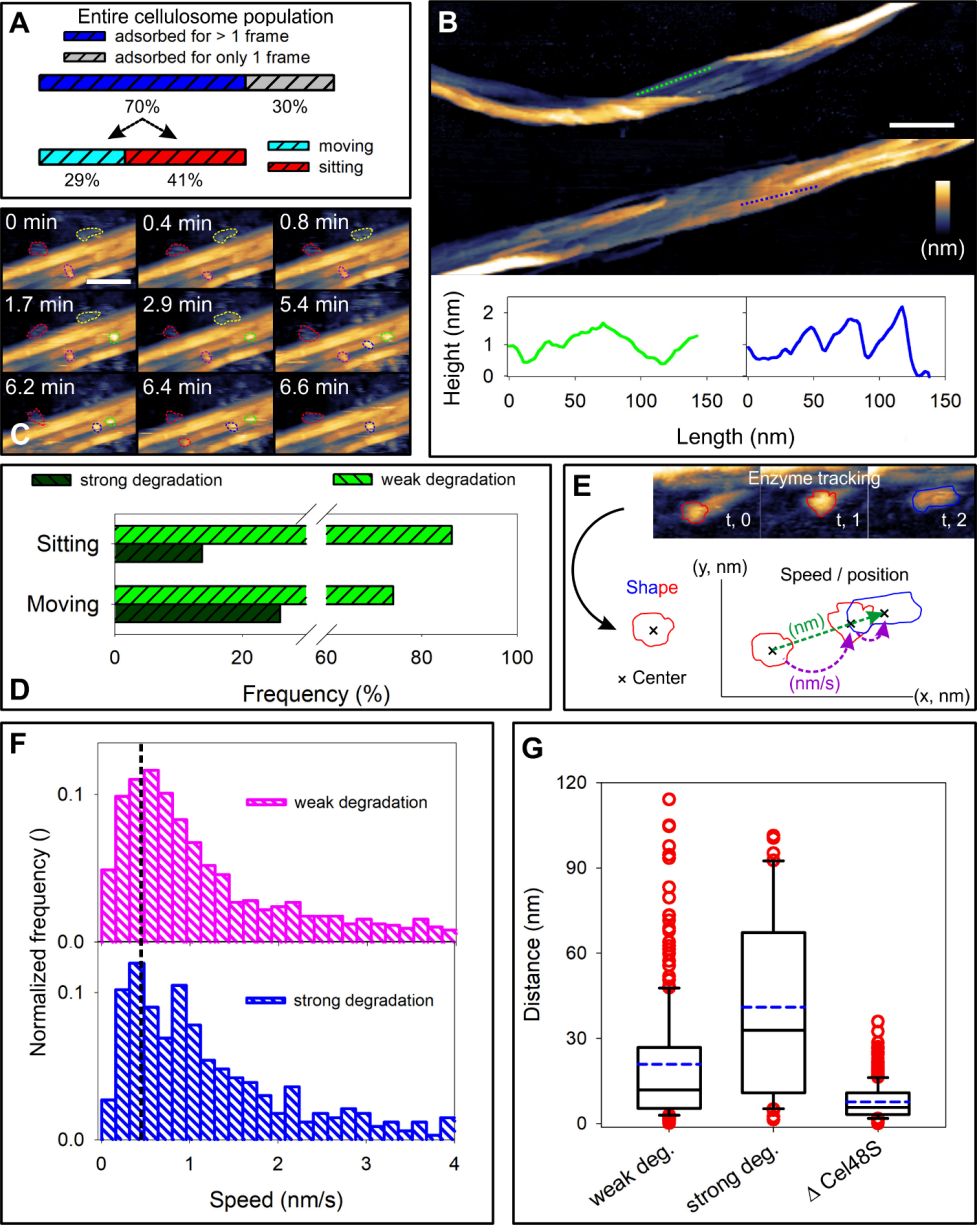
Dynamic behavior of the cellulosomes on cellulose. (A) Subdivision of the cellulosome population (*N* = 512 single molecules) analyzed on cellulose. (B) Example of cellulose fibers with smooth (top) and rough (bottom) fibril surfaces. Height profiles are plotted and leveled along the colored lines to illustrate roughness. (C) Real-time observation of multiple ΔCel48S cellulosomes adsorbing, sitting, and desorbing from the fibril surface. Exemplary individual cellulosomes are marked with a unique color across different time points. (D) Distribution of the cellulosome population according to movement and efficiency of degradation. (E) Workflow of AFM data analysis for automated calculation of the distance traveled, trajectory, and speed of movement. (F) Normalized velocity distribution for the moving cellulosomes, with an average speed at ∼0.5 nm/s indicated (black dashed line). (G) Boxplots depicting the range of distances moved by native and ΔCel48S cellulosomes, with median (solid black line) and mean (dashed blue line) indicated. Scale bars are 100 nm (B) and 50 nm (C). The false color scale used throughout is in panel (A). Height ranges are 18 nm (B, top), 16 nm (B, bottom), and 12 nm (C).

Real-time AFM data revealed subdivision of the cellulosome population according to the length of stay in the adsorbed state and mobility on the cellulose surface. About 30% of the cellulosomes are desorbed in the time between two recorded frames (0.5–5 s, depending on the temporal resolution used; Figure 2A). The remaining 70% (adsorption ≥3 to 7 s) divide into groups of moving (29%) and nonmoving (“sitting”) molecules (41%). Displacement of the cellulosome’s center of mass by 20 nm or more was defined as indicative of movement. The moving cellulosomes were observed to slide unidirectionally along the cellulose surface (Movie S1). Their locomotion was discontinuous, with periods of movement alternating with sitting until the molecule desorbs.

To better understand the salient features of cellulosome motion, we analyzed their relationship with the local nanostructure of the cellulose surface. The single fibers of bacterial cellulose are a superordinate hierarchical nanostructure, consisting of multiple subunits in the form of intertwined microfibrils, as described previously.^32^ These microfibrils were estimated to contain approximately 24 cellulose chains and have periodically occurring defect areas.^32,33^ Defect areas are dislocations between distinct microfibril segments within the same microfibril that can be observed in the height of the AFM image (Figure 2B). The height difference between different segments is about 2 nm (Figure 2B). Correlating cellulose surface properties with a cellulosome movement pattern reveals that the number and spatial distribution of the dislocations control the mobility of the cellulosomes. Directional movement is essentially restricted to “smooth” microfibril regions with long defect-free segments (>100 nm; Figure 2B, green and Movies S1, green and S2a,c,e,f). In “rough” microfibril regions with multiple defects at a shorter distance (≤50 nm, Figure 2B, blue and Movies S1, red and S2b,d), the cellulosomes are unable to move. The observed temporary halting of moving cellulosomes appears to be caused by a larger defect, which is either a coarse dislocation, the end of a fibril, or the beginning of several fibrils nearby. At this stage, the cellulosome either reorients onto a new fibril or remains sitting until desorption.

### Role of Cel48S in Cellulosome Mobility and Substrate Degradation

The enzymatic subunits of the *C. thermocellum* cellulosome comprise chain end-cleaving exocellulases and internal chain-cleaving endocellulases. The main exocellulase Cel48S degrades cellulose processively from the reducing chain end.^34,35^ We hypothesized that Cel48S is critical for the directional movement of the cellulosome. We obtained the fully formed ΔCel48S cellulosome from the relevant gene knockout strain of *C. thermocellum*(36) (Figure S8 and see the SI: Production and Purification of the ΔCel48S Mutant) and showed in AFM experiments that this variant cellulosome (1066 single molecules analyzed) has indeed lost the ability of lateral movement on cellulose. Numerous ΔCel48S cellulosomes are seen adsorbing, sitting, and desorbing from the fibril surface, but none is moving (Figure 2C and Movie S3).

We then assessed how interactions of cellulosomes with cellulose relate to substrate degradation. The rapidly desorbing cellulosomes did not degrade the cellulose. A large portion (87%) of the sitting cellulosomes is bound unproductively, and only the remaining 13% give degradation (Figure 2D). The degradation proceeds mainly in the vertical direction of the fiber (Figure S9). The sitting molecules thus give rise to the distinct fiber-fragmenting mode of cellulose deconstruction that our earlier work^32^ has shown to be characteristic of the cellulosome, as compared to “dispersed” cellulases that do not assemble into stable enzyme complexes^37^ and degrade the cellulose fiber primarily in the lateral direction.^32^ The AFM results reveal furthermore that the ΔCel48S cellulosomes behave similarly as the sitting population of the native cellulosome (Figure S9 and Supporting Data Sets 1 and 2). In solution experiments measuring sugar release from the bacterial cellulose, the native cellulosome is found as 4.5-fold more active than the ΔCel48S variant (Figure S10 and see the SI: Cellulosome Activity Assay). The difference in activity is explained by the moving cellulosomes, which appear to be more productive in substrate degradation than their sitting counterparts. About 26% of the moving cellulosomes show complete deconstruction of the whole microfibril used as a track of their directional movement (Movie S2). The remaining 74% show weaker degradation, often at the limit of detection of AFM. The degradation arguably corresponds to just a few cellulose chains removed from the microfibril surface during the cellulosome’s directional motion.

### Statistical Analysis of Cellulosome Velocity and Travel Distance

We developed a semiautomated MATLAB routine to track precisely the enveloping shape of single molecules over the full sequences of frames collected from real-time AFM measurements (see the SI: Cellulosome Analysis Software and Figures S1–S7). With this highly advanced tailored procedure, multiple cellulosomes present in the same AFM frame could be analyzed comprehensively with respect to the shape change as well as the trajectory and velocity of movement of the center of mass of each single molecule (Figure 2E). We show that the step velocity of the directionally moving cellulosomes exhibits a log-normal distribution with a peak at ∼0.5 nm/s (Figure 2F). The distribution is similar for the cellulosomes featuring strong (S) and weak degradation (W) of cellulose during their movement. Although unable to move, the ΔCel48S cellulosome is not static while sitting. Its position fluctuates due to small (≤20 nm) displacements of the center of mass in a random direction (median velocity = 0.3 nm/s; Figure S11) but effectively remains unchanged overall (Movie S3 and Supporting Data Set 2). The S population of moving cellulosomes surpasses the W population in the median distance traveled by ∼2-fold, 58 versus 27 nm (Figure 2G). The median length of staying on cellulose is longer for the S population (∼33 s) than the W population and the ΔCel48S cellulosome (both ∼21 s; Figure S12).

### Correlation between Cellulosome Shape, Mobility, and Cellulose Degradation

With respect to the nanoscale deconstruction of cellulose performed, the S and W populations of moving cellulosome bear important analogy to dispersed cellulases. The W population resembles processive exocellulases that degrade cellulose by removing single-surface layers of the material one at a time.^25,27,28^ The S population uses a distinct multilayer-processive mode of cellulose degradation that consists of the directed removal of the entire microfibril from the surface. Multilayer-processive degradation was first discovered in our earlier work on cellulases.^33^ To achieve it, endo- and exocellulases engage in the formation of transient clusters of three to four enzymes on cellulose and move directionally as one cluster while degrading the whole fibril. Spatiotemporal coordination of endo-exo activity in the close proximity of the multienzyme cluster was shown as a mechanistic requirement of multilayer-processive degradation.^33^ The (averaged) parameters of speed and distance of directional movement associated with the multilayer-processive degradation differ between cellulase clusters (speed: ∼1.2 nm/s; distance: ∼40 nm)^33^ and the cellulosome (speed: ∼0.5 nm/s; distance: ∼58 nm). The faster processive movement during multilayer degradation might be a factor accounting for the overall ∼5-fold higher specific activity of soluble sugar release that cellulases exhibit on the bacterial cellulose as compared to the cellulosome.^32^ The longer distance of processive movement by the cellulosome might be a consequence of the stable assembly of enzymes into complexes in the cellulosome, contrary to the just transient assembly in clusters by the cellulases. However, such interpretations must be considered tentative.

**Figure 3:**
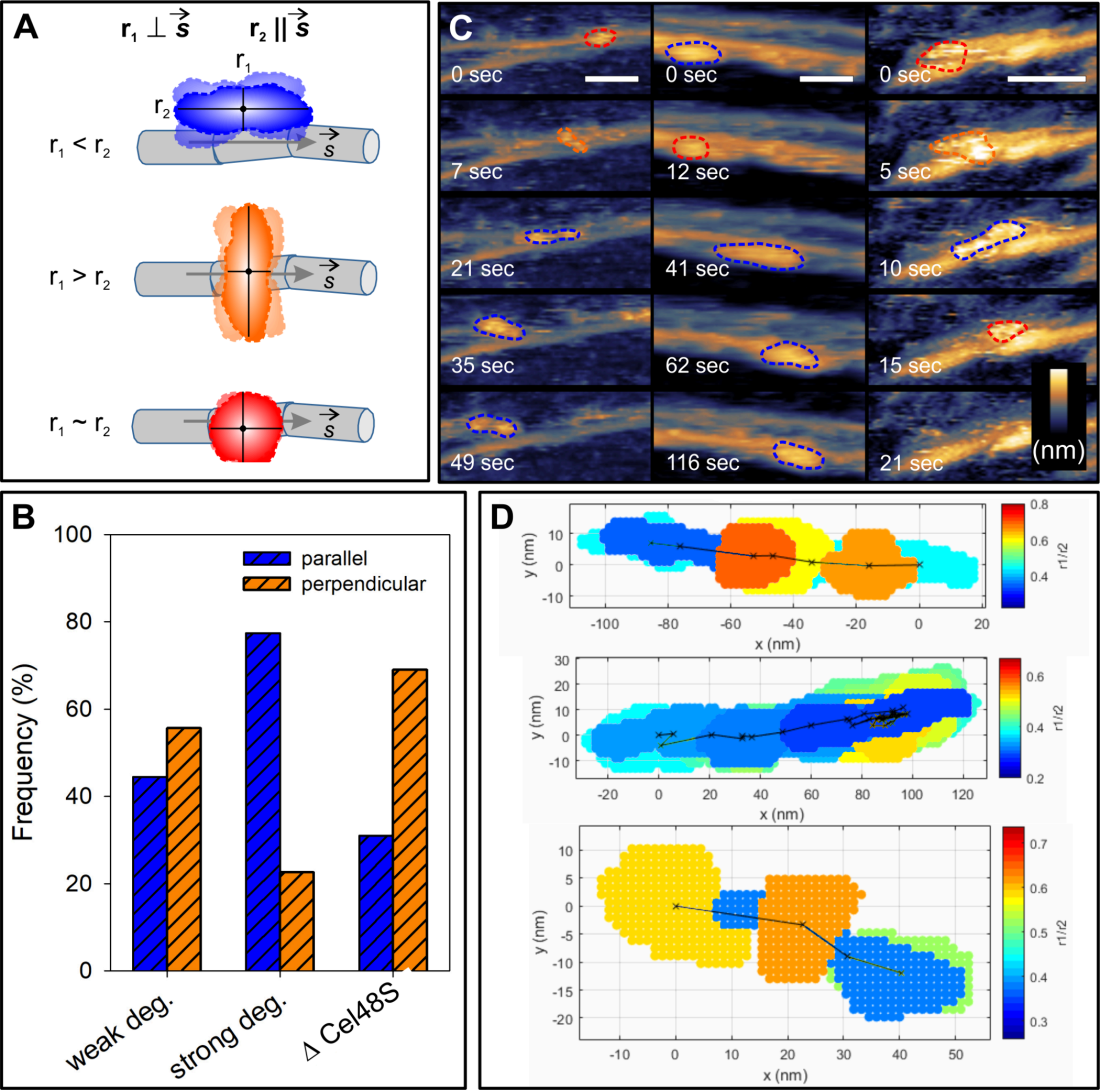
Dynamic changes of the cellulosome shape associated with directional movement and substrate degradation. (A) Quantitative description of the cellulosome shape alignment to the longitudinal axis (s⃗) of the cellulose fiber, based on the ratio of vectors *r*_1_ and *r*_2_. The blue cellulosome is considered aligned (parallel to s⃗) and the other two as unaligned (perpendicular to s⃗). (B) Distribution of aligned and unaligned shapes in populations of native and ΔCel48S cellulosomes. (C, D) Temporal shape changes in three S-type cellulosomes moving along a cellulose fibril while degrading it (C) and their representation along the trajectory of movement (D). The AFM time series from left to right in panel (C) corresponds to the trajectories from top to bottom in panel (D). Scale bars are 50 nm. The false color scale used throughout is in panel (C), and height ranges are 14, 20, and 10 nm from left to right (C).

We thus assessed the molecular shapes of the cellulosome associated with the different styles of degradation. Besides subdivision into elongated and round shapes, we categorize the elongated shapes according to whether they align with the direction of propagation on the fiber (Figure 3A and Figure S13).

Round cellulosomes were considered here as not aligned. Our analysis reveals that the large majority (77%) of S population cellulosomes adopts elongated-aligned shapes (Figure 3B). In the W population, the frequency of elongated-aligned shapes decreased to 44% (Figure 3B). In the ΔCel48S cellulosome population, any molecular shape including the degree of alignment is equally probable (Figure 3B). Analyzing the aligned cellulosomes of the S population as they move directionally, we observe that their shapes extend and compress periodically, as in a macromolecular crawl of a protein complex that involves elastic switches between elongated and compact arrangements of the scaffolded subunits (Figure 3C,D and Movie S4 and Supporting Data Set 3). The nanomechanics of cellulosome movement thus appears to consist of multiple Cel48S subunits propagating forward while pulling along as cargo to other enzymatic subunits that do not move independently but are elastically tethered to them via the scaffold protein ( Supporting Data Set 3).

## Discussion

**Figure 4:**
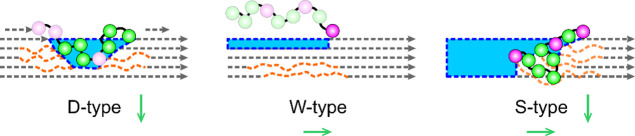
Schematic representation of directional movement of the cellulosome as a molecular motor and its relationship with mode and efficiency of cellulose degradation. Cellulose chains, with the direction to the nonreducing end marked, are organized into crystalline material (gray), with regions of local disorder indicated in orange. The engagement of Cel48S exocellulase (magenta) and endocellulase subunits (green) in the particular type of degradation is indicated by the corresponding circles shown as filled (engaged) or transparent (not engaged of necessity). The cellulose material removed is shown in blue, where area size indicates the assumed relative importance of the particular degradation type to the total degradation. The multilayer S-type degradation of moving cellulosomes involves the exo- and endocellulase subunits fully engaged to degrade the whole fibril in lateral and vertical directions (green arrows), and it is crucial for the full native activity of the cellulosome. See the text for further discussion.

We conclude from the evidence shown that the cellulosome transforms dynamic changes of its quaternary conformation into a versatile function in substrate degradation. Random fluctuations of conformation occur in cellulosomes sitting on a cellulose surface too rough for directional movement. Conformational flexibility, well documented in studies of the cellulosome in solution,^38,39^ is likely critical in these cellulosomes to accommodate the locally emerging nanomorphologies of the cellulose surface during substrate degradation focused in the vertical direction of the fiber (D-type; Figure 4). Directional movement of the cellulosomes on smooth cellulose surfaces requires mechanochemical coupling of catalysis and motion and is strictly dependent on the processive exocellulase Cel48S. The moving cellulosomes of both W- and S-types work as molecular motors that operate by a Brownian ratchet mechanism. They rectify random cellulosome motions on the cellulose into directed movement via coupling to the Cel48S hydrolysis of cellulose chains. Directional preference is explained by a “burnt bridge” model of chemomechanical movement^24,40^ where the Brownian ratchet motor degrades its linear track on propagating forward, so diffusion backward on the same track is impossible. We interpret multilayer degradation of cellulose fibrils by S population cellulosomes to arise from Cel48S exocellulases that enabled synergy with endocellulases in the aligned-elongated conformation of the cellulosome (Figure 4). The less efficient (single-layer) degradation by W population cellulosomes is ascribed to Cel48S exocellulase being unable to establish endo-exo synergy. A possible reason is that the local nanostructure of crystalline cellulose is not well accessible for endocellulase chain cleavage or it prevents multiple Cel48S subunits from attacking simultaneously parallel tracks of the cellulose chain on the fibril surface (Figure 4).

The biological consequences of cellulosome motor activity are of great interest. Cellulosome efficiency in fiber deconstruction critically relies on the concerted work of moving motors and sitting drillers. Crystalline fiber fragments released by the drillers will require the motors for complete degradation. Our single-molecule evidence reveals limitations in cellulosome efficiency due to nonproductive adsorption. Engineering of the enzyme and substrate should involve the relative amount of S-type motors in the cellulosome population on cellulose as a highly promising target. The phenotypic observation that *C. thermocellum* detaches cellulosomes from its cell surface during growth on cellulose^6,41,42^ might involve the biological rationale of enabling the cellulosome to optimum motor activity to achieve efficient and complete substrate utilization. We speculate that by coupling to cellular mechanosensing, the movement of cell-associated cellulosomes on cellulose could play a role in the regulation of cell surface localization and density of the cellulosomes.^12,41−43^ Engineered cellulosome motors^13,20,44−46^ able to transport scaffolded cargo along rails of cellulose fibril are inspirational for molecular nanotechnology.^47−50^

## Supporting Information Available

Detailed description of the materials and methods involved in the preparation, purification, and characterization of bacterial cellulose, native cellulosomes, and ΔCel48S mutant cellulosomes; sample preparation for AFM observations; AFM operation, image processing, and data analysis; implementation of step 1 in MATLAB (Figure S1); schematic representation of step 2.1 (Figure S2); schematic representation of step 2.2 (Figure S3); schematic representation of step 2.3 (Figure S4); schematic representation of step 2.4 (Figure S5); schematic representation of step 2.5 (Figure S6); example of the ID finding step in the routine (Figure S7); SDS-PAGE analysis of the purified wild-type and mutant cellulosomes (Figure S8); AFM snapshots of cellulosomes degrading bacterial cellulose fibers (Figure S9); comparison of the conversion rates of native and mutant cellulosomes (Figure S10); boxplot depicting the range of velocity observed for ΔCel48S cellulosomes (Figure S11); comparison of the typical residence times for cellulosomes engaged in different degradation processes (Figure S12); shape tracking example (Figure S13) (PDF)
Real-time observations of the cellulosomes from *C. thermocellum* on bacterial cellulose (Movie S1) (MOV)
Prominent examples of massive volume degradation of fiber parts by the cellulosomes (Movie S2) (MOV)
Real-time observation of ΔCel48S cellulosomes acting on bacterial cellulose (Movie S3) (MOV)
Snapshots of real-time AFM observations of representative S population cellulosomes (Movie S4) (MOV)
Supporting Data Set 1 (ZIP)
Supporting Data Set 2 (ZIP)
Supporting Data Set 3 (ZIP)

## Supplementary Material

cs3c05653_si_001.pdf

cs3c05653_si_002.mov

cs3c05653_si_003.mov

cs3c05653_si_004.mov

cs3c05653_si_005.mov

## ABBREVIATIONS

AFM: atomic force microscopy
HOPG: highly ordered pyrolytic graphite
SI: Supporting Information
