# Supplementary material for: Mechanochemical Coupling of Catalysis and Motion in a Cellulose-Degrading Multienzyme Nanomachine
Source: ACS Catal. 2024 Feb 6;14(4):2656–63. doi: 10.1021/acscatal.3c05653 (PMC10877591; doi:10.1021/acscatal.3c05653)
Supplement: Supplementary file 1 — cs3c05653_si_001.pdf [file cs3c05653_si_001.pdf]

Supporting Information

Mechanochemical coupling of catalysis and motion in a cellulose-degrading multienzyme nanomachine

Krisztina Zajki-Zechmeister[1], Manuel Eibinger[1], Gaurav Singh Kaira[1,2], and Bernd Nidetzky[1,2,]*

[1]Institute of Biotechnology and Biochemical Engineering, Graz University of Technology, Petersgasse 10-12/1, 8010 Graz, Austria.

[2]Austrian Centre of Industrial Biotechnology, Petersgasse 14, 8010 Graz, Austria.

Corresponding Author

*Correspondence should be addressed to B.N. (bernd.nidetzky@tugraz.at)

Methods

Unless stated, all chemicals were of the highest purity available from Carl Roth + Co KG (Karlsruhe, Germany).

Preparation and isolation of bacterial cellulose fibers

Acetobacter xylinum (46602, DSMZ-German Collection of Microorganisms and Cell Cultures GmbH, Braunschweig, Germany) was used to produce bacterial cellulose films as described in detail in our earlier work.[1] 2.0 mL of the preculture was inoculated in 200 mL sterile yeast peptone mannitol medium (360, DZM) in 500 mL Erlenmeyer flasks and incubated statically at 30 °C for 14 days. Two cellulose films were synthetized at the gas-liquid interface during this time. The first film was removed after 7 days to vacate space for the second film, which formed during the remaining 7 days. The second film was harvested and cleaned for further usage. Cleaning was performed by rinsing the film with ultrapure water and incubating it with 1 M sodium hydroxide for 24 h, at 4 °C under magnetic stirring. Finally, to remove residual biomass, media components and sodium hydroxide, the film was repeatedly rinsed with ultrapure water until the pH reached 7.0. Subsequently, the cellulose film was disrupted using a conventional hand mixer in ultrapure water for 5 min, resulting in a highly diluted cellulose suspension (<0.01 g/L). To detangle the fiber bundles further into individual fibers, ultrasonic homogenization was performed using a Sonic Dismembrator (Fisherbrand Model 505, Fisher Scientific GmbH, Im Heiligen Feld, Germany) operated with a 6A/4V titanium alloy probe head (Fisherbrand™ FB4406, Fisher Scientific GmbH). A 2 sec pulse at 40 % intensity was followed by a 5 sec pause for a total pulse duration of 20 min. The suspension was cooled in an ice bath during the homogenization process. The resulting suspension containing individual fibers, gently released from the three-dimensional fiber network from the original film was, stored at 4 °C.

Preparation of cellulosomes

Clostridium thermocellum (ATTC 27405[2,3]) was grown in GS-2 media on microcrystalline cellulose as reported previously.[4] The cultivation was done in sealed 100 mL flasks filled with 50 mL of medium containing Avicel PH-101 (10 g/L) as carbon source. Anoxic conditions were monitored by using Resazurin as a redox indicator. Cultivation was done for about 72 h, at 55 °C with shaking (100 rpm). Note, that after 72 h, a majority of the carbon source had been typically dissolved. The fermentation broth was cleared by two-fold centrifugation at 4500 g for 20 min at 4 °C, Eppendorf, 5804 R. The supernatant containing the cellulosomes was filtered

through a disposable syringe filter with a pore size of 0.2 µm (Minisart, Sartorius, Göttingen, Germany) and the buffer was exchanged to 30 mM MOPS buffer with pH 7.0, containing 100 mM NaCl and 10 mM $CaCl_2$. This was done using disposable centrifugal concentrators with a molecular mass cut-off at 300 kDa (Vivaspin Turbo 15, Sartorius). Cellulosomes were purified using size-exclusion chromatography using a preparation grade column (HiLoad® 16/60 Superdex 200, GE Healthcare, Little Chalfont St. Giles, UK) as reported previously [4]. Fractions containing cellulosomes were pooled, concentrated (~200 mg/L) and stored at 4 °C. The protein concentrations were measured using the Bradford assay with ROTI®-Nanoquant calibrated using BSA as standard.

Production and purification of the ΔCel48S mutant

The *Δcel48S* mutant strain of *Clostridium thermocellum* was received from Prof. Lee R. Lynd (Department of Biological Sciences, Dartmouth College, Hanover, U.S.A.). Primary cultures of the strain were grown in 50 mL uracil supplemented (40 µg/mL) GS-2 media using 100 mL bottles at 55 °C and 80 rpm. The primary culture was used to inoculate (4 %) the main culture (400 mL uracil supplemented GS-2 media in 500 mL bottles). The main culture was cultivated, similar to the native strain (see *Preparation of cellulosomes*) for 72 h, at 55 °C and 80 rpm using Avicel (10 g/L) as the carbon source. After the cultivation, the culture medium was centrifuged twice (4500 g for 20 min at 4 °C, Eppendorf, 5804 R) to remove the residual pellet. The supernatant was concentrated and buffer exchanged to 30 mM MOPS buffer (pH 7.0) containing 100 mM NaCl and 10 mM $CaCl_2$, using a 100 kDa centrifugal concentrator tube (Vivaspin®, Sartorius, Germany). The protein solution was filtered using 0.2 µm PES syringe filter (Sartorius, Germany) and the ΔCel48S cellulosome was purified using size exclusion chromatography (HiLoad® 16/60 Superdex 200, GE Healthcare) similarly as the native cellulosome. The purified protein was analyzed using SDS PAGE (Fig. S8). The protein concentrations were measured using the Bradford assay with ROTI®-Nanoquant calibrated using BSA as standard.

Cellulosome activity assay

Hydrolysis reactions were performed in a volume of 1 mL in 1.5 mL Eppendorf tubes in freshly prepared 30 mM sodium acetate buffer, pH 5.5, supplemented with 100 mM NaCl, 10 mM $CaCl_2$, 10 mM cysteine and 2 mM EDTA. Bacterial cellulose fibers (1.0 mg/mL) were hydrolyzed using cellulosomes (2.0 µg/mL) at 55 °C, 500 rpm. All reactions contained 2 µg/mL beta-glucosidase and were carried out in duplicates. At defined times samples were aliquoted and the reactions were stopped by adding NaOH to a final concentration of 100 mM. Thereafter, samples were centrifuged, and the supernatant was assayed for total glucose using a using

commercially available kit (D-Glucose HK assay kit, Megazyme, Dublin, Ireland). The bacterial cellulose degradation (%) was calculated using the glucose release per total anhydrous glucose content of bacterial cellulose (Fig. S9) as described recently [1].

Atomic Force Microscopy – Set-Up of the in-situ visualization of the cellulosome

Atomic force microscopy (AFM) measurements were performed with a commercially available atomic force microscope (Dimension Fast Scan Bio, Bruker, Billerica, MA, USA). The controller (Nanoscope V, Bruker) was operated with the associated software (Nanoscope 9.2, Bruker). All measurements were performed in tapping mode in liquid environment using suitable probes (Fast Scan DSS, Bruker) with nominal frequency, spring constant and tip radius of 110 kHz, 0.25 N/m and 1 nm, respectively, as described in our earlier work [5]. Individual cellulose fibers were immobilized on a freshly cleaved highly oriented pyrolytic graphite crystal (HOPG, grade I, SPI supplies) by incubating 1 cm^2 surface with 300 µl of highly diluted cellulose suspension (<0.01 g/L) for 15 min. The crystal was rinsed with deionized water and residual droplets were removed by spaying carbon dioxide on the surface for 3 sec. The crystal was quickly mounted onto the liquid stage of the AFM and wetted with 250 µL buffer to minimize contamination of the surface. The buffer droplet was already the liquid environment in which the AFM scan head including the already mounted probe was immersed. Once completely immersed, the light microscope camera setting was set to "liquid". The laser-detector alignment was performed with care, until the incoming signal at the photodiode was at least 1.5 V. A preliminary "Auto tune" was performed to adjust the excitation frequency of the probe appropriately. The scan head was carefully driven down until a surface feature (either HOPG edge or some impurities) was fully focused on the camera. Prior to tip-surface engagement, the stage holding the HOPG crystal was heated with a temperature controller (Bruker) to 35 °C for an equilibration time of 20 to 40 min. The equilibration was finished, once the spatial position of the signal at the photodiode was stable. The tip was engaged using the "smart engage" setting. 30 µm × 30 µm areas were scanned systematically until a fiber was found that was sufficiently attached to the HOPG ground and not in the direct vicinity of other fibers. To ensure that the subsequent image correction works as well as possible, care was taken that the fibers were laying diagonally in the scan area to prevent that entire horizontal pixel lines would consist only of fiber pixels. After thorough measurement of the fiber the cellulosome was injected into the liquid environment. This was done by carefully pipetting 10-60 µL cellulosome (15 - 79 mg/L) in 10 µL portions. The injection in small portions was chosen to avoid creating too big turbulences in the liquid that could cause the fiber to drift away from the scan area. The fiber was continuously measured for at least 2 h or until it was deconstructed

completely. For observations that exceeded 3 h of measurement time it was necessary to inject additional buffer due to evaporation. All buffer and enzyme preparations were preheated to 35 °C prior to injection.

Atomic Force Microscopy – Measurement parameters

Parameters were selected and adapted as described in our earlier work [5]. All frames were captured with 6 channels: topography, phase and amplitude in trace and retrace direction. As soon as the tip was in contact with the surface, a surface tune was performed to readjust the exiting frequency. Regarding the force load on the sample, the Amplitude Setpoint was set to ~70-90 % of the free amplitude. If the phase image showed instable regions even at low Set points, the Drive Amplitude was increased in small steps. Once a sufficient combination of Set Point and Drive Amplitude was found, care was taken to keep these parameters constant throughout the measurement. However, in the case of any drift of the z-piezo or shift of laser signal at the detector, it was adjusted accordingly. The Integral Gain was continuously set to the highest possible value at which no resonance was yet visible in the amplitude image. The Proportional Gain was set to a value 3-5 times greater than the Integral Gain. The vertical deflection of the tip was reduced, by setting the Z-range of the scanner to half of its maximum value.

In contrast to our earlier cellulosome experiments[1,4], the focus was on dynamic movement of the enzyme complex on top of the fiber surface instead of the topological changes on the fiber itself. A challenging balance had to be found between highest possible time resolution and minimal influence on the flexible and soft complex. Additionally, the time resolution had the extra requirement to be at least 0.14 frames/sec, so that individual cellulosomes could be identified visually across different frames (is it the same cellulosome, but moved to another position or is it a newly adsorbed one, whilst the other desorbed).

To optimize time resolution, scan sizes were chosen as small as possible while still depicting relevant parts of the fiber. This resulted in small scan areas (45000 $mn^2 \leq$ scan area $\leq$ 100000 nm^2). The scan window was set to a rectangle with an aspect ratio of at least 1:3 and maximum 1:5 to minimize measuring unnecessary background pixels. The scan speed was increased until either tracking errors or other measurement artefacts could no longer be corrected by changing the feedback parameters or until the scanning seemingly resulted in disturbance/obscureness of the cellulosomes. To fully exhaust all available options to enhance the speed even further, occasionally the amount of measurement points was reduced by choosing larger pixel step sizes or by skipping ever other horizontal measurement line. Note, that this results in images with

non-square pixels. This was considered in the data correction. With this, a speed of up to 3 frames/s was accomplished. Spatial resolution was usually 2 nm/pixel or better.

Data correction and video construction

Preprocessing of AFM images for pixel-based calculations as well as for generation of time-lapse movies was performed using a self-developed MATLAB routine, thoroughly described in our previous work.[5] Briefly, the routine involved 4 main steps: object masking, data correction, scaling and drift correcting. Object masking was done by identifying edges and surfaces of objects (enzymes, fibers) and background (HOPG) using user-defined gradient and median parameters. Data correction for the height channel involved correcting for tilt and mismatched baselines of rows, and scaling was done by setting the lowest value of each image to zero and then scaling all images to one user-defined maximum value. The phase and amplitude data were not manipulated and were only processed with regards to false-color scaling. Drift correction was performed by comparing a user-set reference image to all other images in the sequence and shifting each image to best match the reference image. The final processed data sets were exported as .png images for movie generation and as 2D matrices for further analysis. Supplementary movies were created with Davinci Resolve (Version 17.2.1, Blackmagic Design, Port Melbourne, Australia).

Cellulosome analysis software

To track the movement and shape of individual cellulosomes, a semi-automated 3-step MATLAB (developed in R2017b, Version 9.3.0.713579; MathWorks, Natick, Massachusetts, USA) routine was developed. In the first step, all frames of a sequence were loaded into the program in such a way that it was possible to scroll back and forth between the individual images. In addition, the height, phase and amplitude channel were simultaneously displayed for each frame. It was ensured that all three channels show the same section of the frame when zooming or moving within it in order to ensure optimal accuracy in the masking process (Fig. S1). For outlining, the position of the mouse cursor was read out and, inspired by the masking option in Gwyddion, the outline of the structure of interest could be traced pixel by pixel with the tip of the mouse pointer. Once the boarder of all cellulosomes were masked in a frame, the same procedure is repeated with the next frame. Numerically, masked pixels were assigned a value of 1 and 0 otherwise. To mitigate systematic deviations in the masking style caused by multiple individuals, a single person conducted the frame evaluations. Undoubtedly, tasks involving object masking in AFM evaluations exhibit a degree of inherent uncertainty. Our previous studies have demonstrated that human biases contribute to an approximate difference of 20% or less in object masking, leading to variations in identifying pixels belonging to the

object versus the background. [5] However, it is important to emphasize that this 20% uncertainty does not alter the foundational assertions of our research, which remain firmly established and unequivocal. The second step was to assign the correct spatial dimension to the drawn outlines, to identify where the "inside" and "outside" of the envelopes of each cellulosome are, and to fill them in to also determine the area of the cellulosomes. It was necessary to already differentiate between cellulosomes within one frame, especially for the cases where several cellulosomes share the same pixels (touch each other on one side). All of this was achieved by an automated routine with 5 substeps: Step 2.1 was to identify interconnected structures. For this each pixel of each masked frame was systematically checked. If the pixel entry was 0, the program jumped to the next pixel, if it was 1, an enveloping cellulosome pixel was found (red circle in Fig. S2 A). In this case, the immediately neighboring pixels were read. Since the mark is always a closed circle, at least 2 of the neighboring pixels must also be involved in the connected structure (Fig. S2 A). As soon as the neighboring enveloping pixel is found, this step is repeated until all pixels within one structure were identified (Fig. S2 B). With the indices of the envelope pixels stored, the next step is to differentiate between pixel placed inside and outside the envelope (step 2.2). To achieve this, the image was first expanded by one pixel at each frame edge. This new row of pixels is safely outside a cellulosome structure and serves as a starting point. Starting at the first pixel, its horizontal and vertical, but not diagonal, neighbor pixels are read out. If they have the same value, they are stored as "background pixels" and the algorithm hops to the next pixel where it repeats the query. This is executed until all background pixels have been identified (blue area in Fig. S3 A). Now the image is simply inverted and all pixels that are not background must be part of at least one cellulosome structure (orange area in Fig. S3 B). To check whether a found structure consists of several touching structures, the fact, that there has to be a masked boarder separating them (step 2.3). The masked boarder is subtracted from the total area of the structure found in step 2.2 (Fig. S4 A). If a structure consists of multiple touching cellulosomes, the remaining area is separated now (Fig. S4 B). If several cellulosomes are marked in one frame, it is important to assign the different area pixels to the respective cellulosomes (step 2.4). For this purpose, the algorithm from step 2.1 is used again, where for each pixel, its horizontal, vertical and diagonal neighbors are read out. If they have the same entry as the currently observed pixel, they are assigned to the structure (Fig. S5 A). Once all pixels have been assigned to a structure, the algorithm jumps to the next cellulosome and repeats the process until all pixels have been assigned (Fig. S5 B). To get back the edge of the structures and to assign it to the correct cellulosome, each area-structure is enlarged by 1 pixel per step (step 2.5). This enlargement happens only horizontally and vertically (not

diagonally) and only to pixels which were previously marked as total cellulosome structure in step 2.2. This step is repeated until all previously found pixels have been assigned again (Fig. S6 A,B). Finally, the marked cellulosomes were found, all pixels were correctly assigned, and the case of "shared" pixels was also handled (Fig. S6 C). The conversion factor from pixel side length to nanometer was read directly form the AFM raw data files, thus, it was possible to read out their perimeter, and area (Fig. S6 D) in nm and nm^2 respectively. The final step 3 was to assign a unique identity (ID) to the respective cellulosomes in order to track their movement across multiple frames. For this purpose, the just found structures were reloaded onto the AFM sequence and the user could assign a unique number to a structure by clicking on it (Fig. S7). Scrolling through the entire sequence allowed to track each cellulosome individually until its desorption. (Fig. S7 A,B,C). To keep track which cellulosome was already assigned an ID, the following color scheme was implemented: Green – current ID, red – no ID, blue – assigned ID which is not the current one (Fig. S7 D).

In silico size estimation of celllulosomes

To determine whether there is an average presence of nine cellulases within the cellulosome, we conducted *in silico* examinations of various spatial configurations of cellulases, using exocellulase Cel48S (PDB code 5yj6) as an exemplar. These simulations were conducted following the methodology previously outlined in our work.[5]

PDB files were generated using Pymol (Version 4.6.0) and visualized with the BioAFMViewer.[6] The AFM images generated with BioAFMViewer were visually compared to in vivo measured AFM height images. Settings were selected to match the average tip's geometry (Fast Scan-DSS, cone angle 15°, tip radius: 1 nm) and measurement conditions (scan step: 1-2 nm).

Shape calculations

The two vectors for each shape $\vec{r}_1$ and $\vec{r}_2$ were determined by calculating the eigenvalues and eigenvectors of the covariance matrix of each shape. For each time point, the position of all relevant pixels are stored in a matrix P. All components in x direction are subtracted by the mean $\bar{x}$ value, resulting in $\vec{x}_m$. The same is done for the y values, giving $\vec{y}_m$.

$$P = \begin{pmatrix} x_1 & y_1 \\ x_2 & y_2 \\ \vdots & \vdots \\ x_n & y_n \end{pmatrix} \tag{1}$$

$$\vec{x}_m = \begin{pmatrix} x_1 \\ x_2 \\ \vdots \end{pmatrix} - \bar{x}, \qquad \vec{y}_m = \begin{pmatrix} y_1 \\ y_2 \\ \vdots \end{pmatrix} - \bar{y} \tag{2}$$

From this, the covariance matrix can be calculated, with *n* being the number of elements in $\vec{x}_m$.

$$C = \begin{pmatrix} \vec{x}_m^T * \frac{\vec{x}_m}{n} & \vec{x}_m^T * \frac{\vec{y}_m}{n} \\ \vec{x}_m^T * \frac{\vec{y}_m}{n} & \vec{y}_m^T * \frac{\vec{y}_m}{n} \end{pmatrix} \tag{3}$$

The eigenvectors of this matrix give the direction of the short and long axes ($\vec{r}_1$ and $\vec{r}_2$, respectively) and the eigenvalues their lengths in every frame (Fig. S12).

Supporting Figures

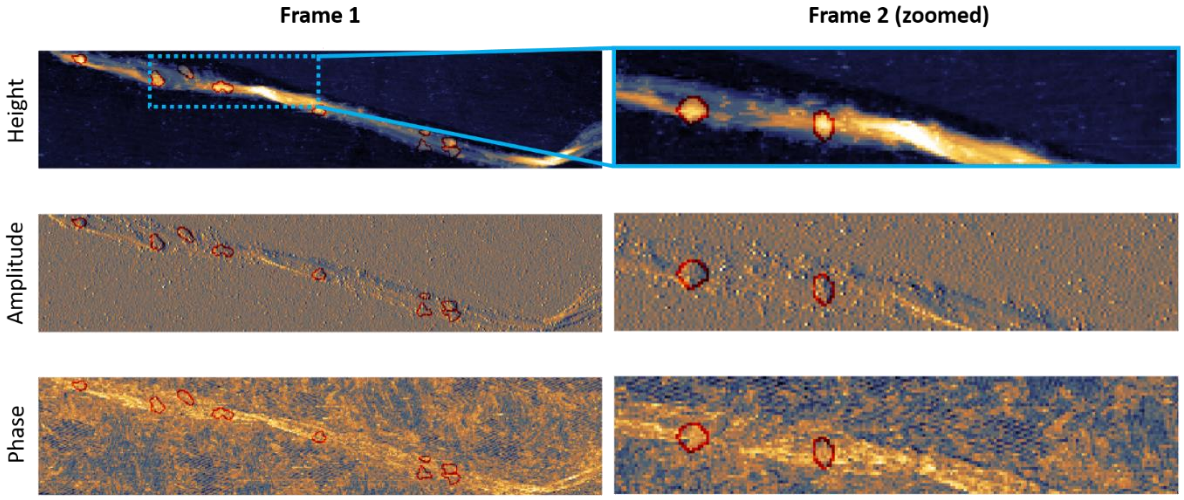

Fig. S1. Implementation of step 1 in MATLAB.

For every frame, height, amplitude and phase channels are visible. Cellulosome borders can be traced with the cursor tip(left). Scrolling through the sequence and zooming within the frame was possible (right).

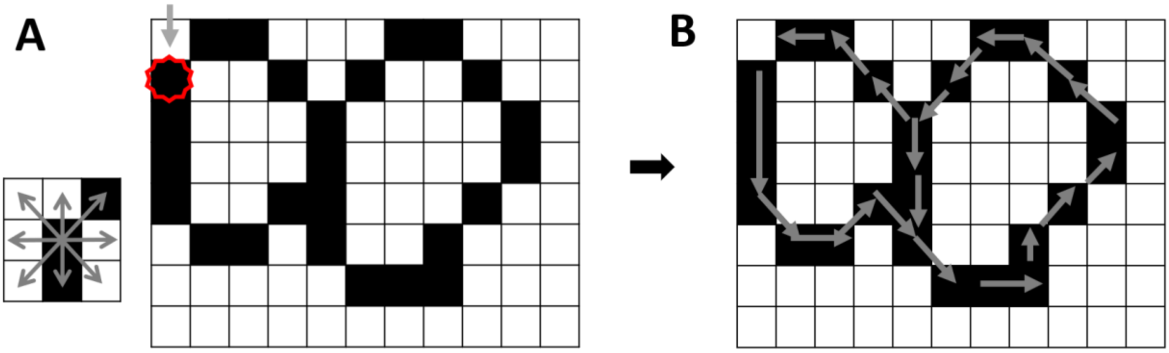

Fig. S2. Schematic representation of Step 2.1.

(A,B) As soon as a marked pixel is found, the neighboring pixels are read out (A). The alorithm hops to the next edge pixel it can find and repreates the step, until all edge pixels are found (B).

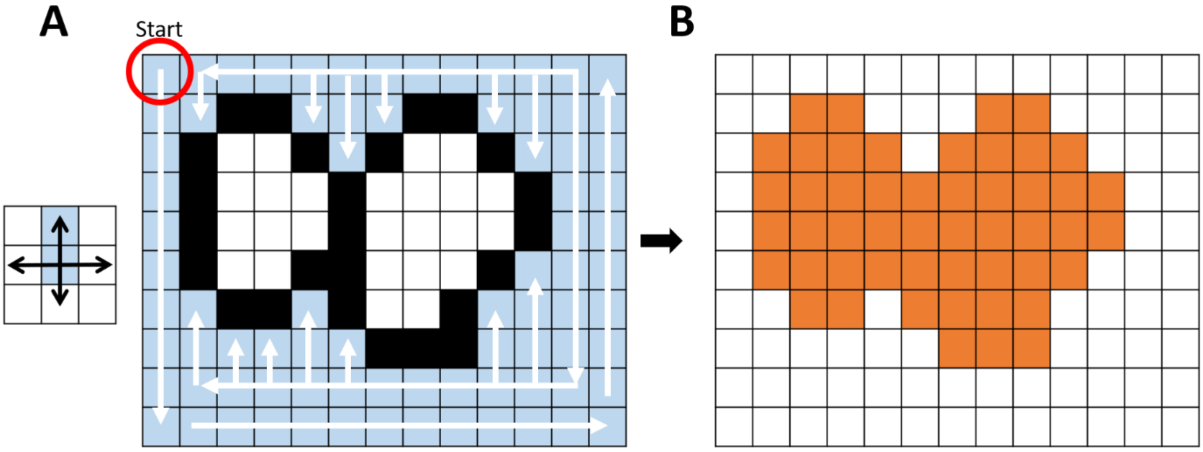

Fig. S3. Schematic representation of step2.2.

(A) After expanding the image for 1 pixel row on all edges, for every pixel the horizontally and vertically positions neighbors are checked if they have the same value. This is done until all background pixels are found.

(B) Inverting the image results in all the pixels which were not classified as background, thus being cellulosome pixels.

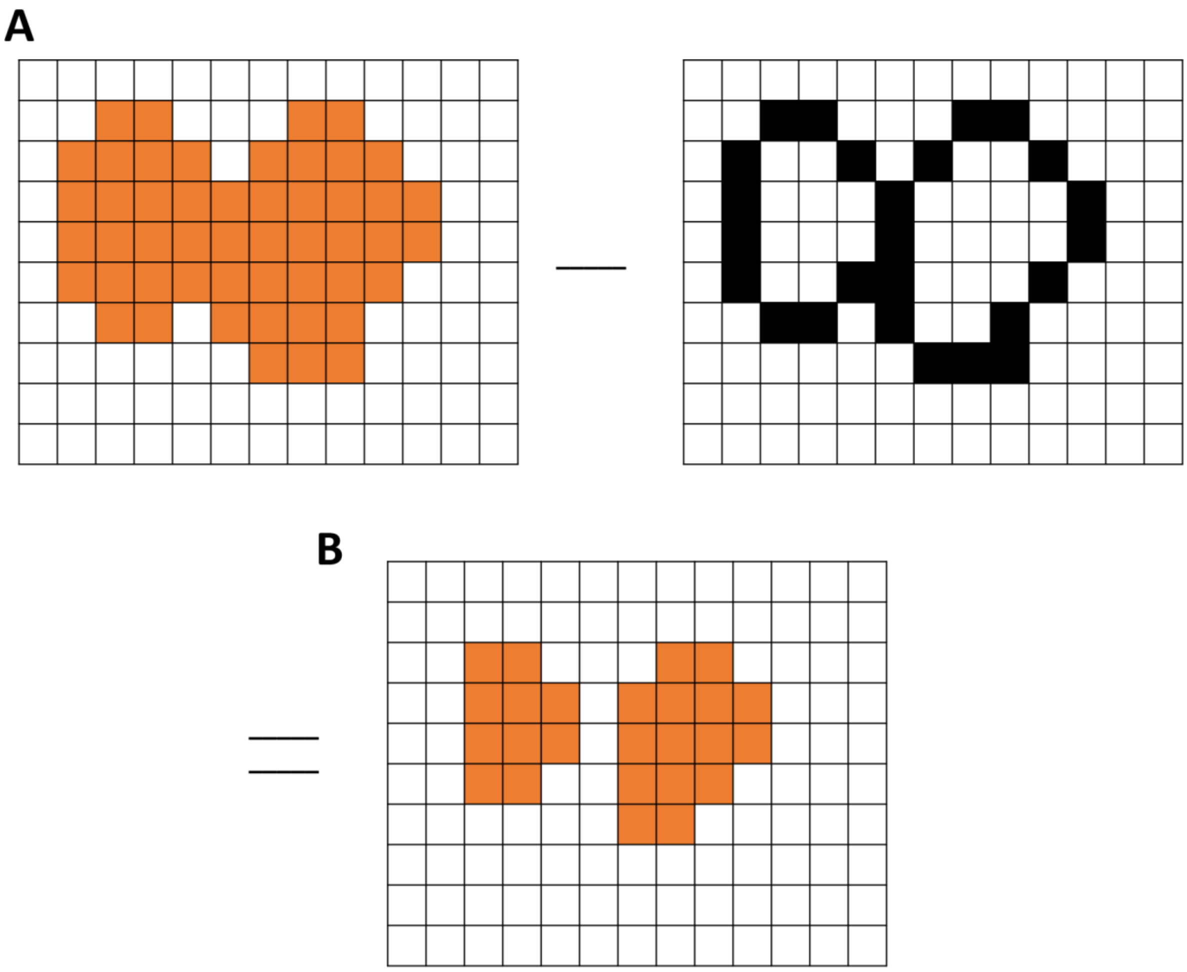

Fig. S4. Schematic representation of step 2.3.

(A,B) Subtracting the previously found edge pixels from the total cellulosome pixels (A), results in only the are pixels of each structure (B).

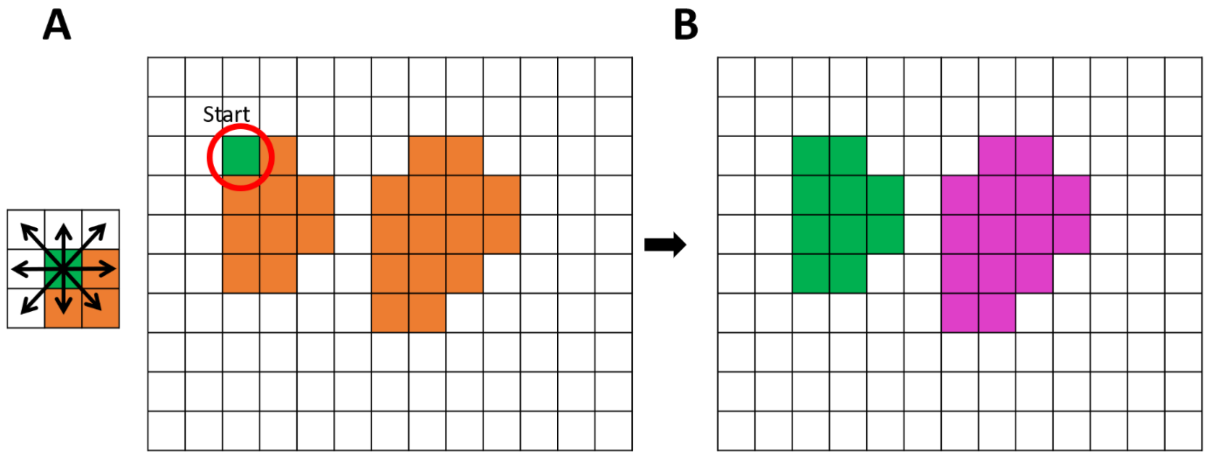

Fig. S5. Schematic representation of step 2.4.

(A) The area pixels of one structure are systematically queried for their neighbors as in step 2.1, until all pixels are assigned to one structure.

(B) After that the algorithm hops to the next structure and repeats the query.

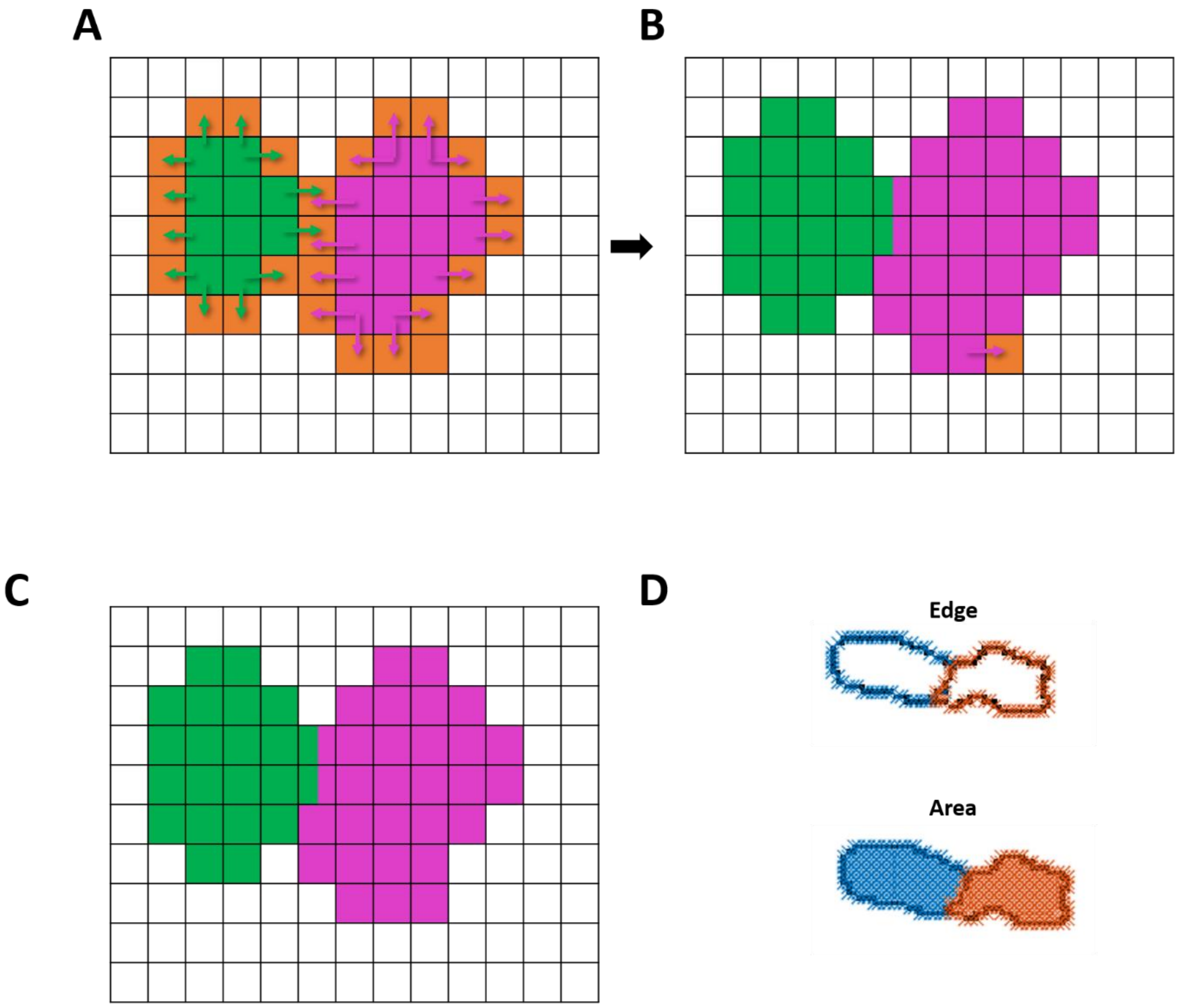

Fig. S6. Schematic representation of step 2.5.

(A) Enlargement of every structure by one pixel horizontally and/or vertically, but only on positions which were previously marked as total cellulosome structure.

(B) This was repeated until all relevant pixels are assigned.

(C) This results in the correct cellulosome structures, regardless of whether there are split pixels or not.

(D) Example for actual structure that was found.

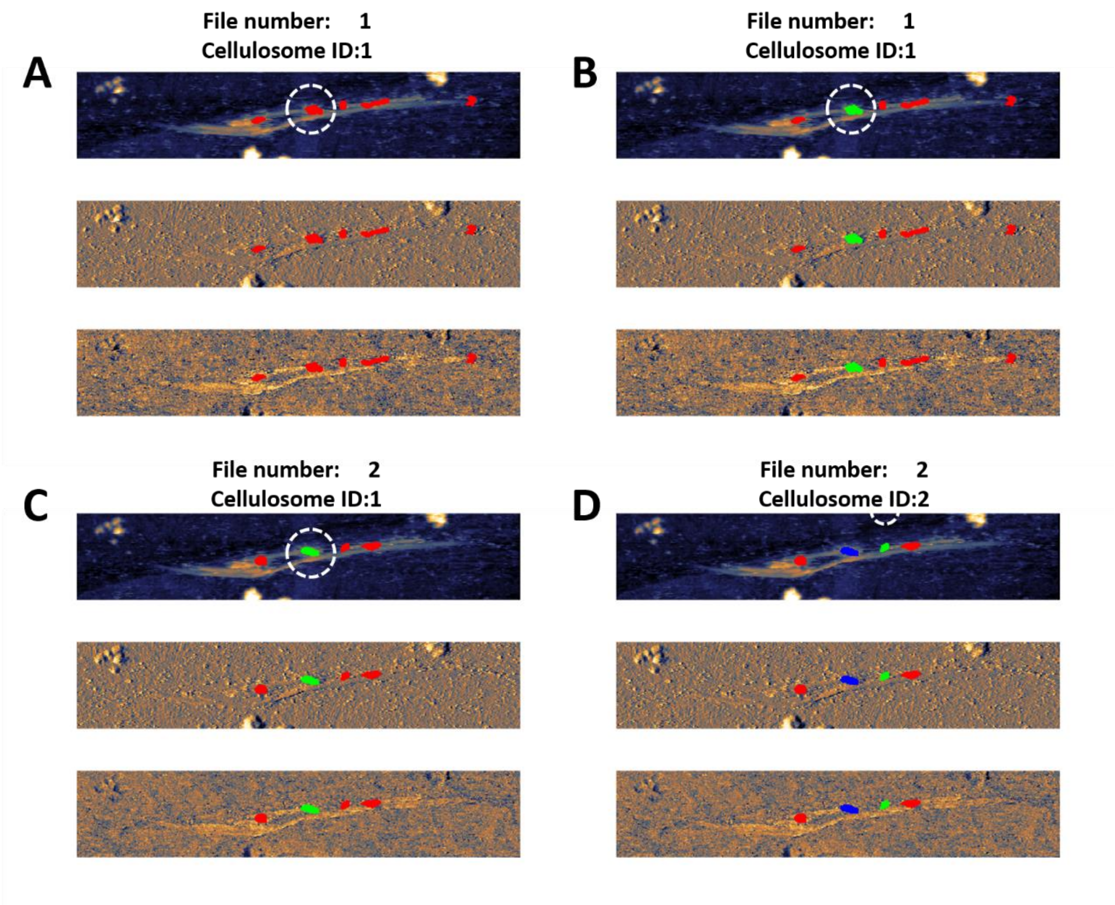

Fig. S7. Example of the ID finding step in the routine.

(A) The automatically found cellulosome structures from step 2 were reloaded onto the AFM images.

(B) Per mouse click a cellulosome was selected and given the ID “1”. The selection was shown by another (green) coloring.

(C) Scrolling to the next frame allowed to select the same cellulosome again.

(D) If the cellulosome desorbed in the next frame, the ID counter was increased (to “2”), the already assigned cellulosomes changed color again (blue) to indicated, that this cellulosome was already assigned previously and the next cellulosome could be selected, now with ID “2”.

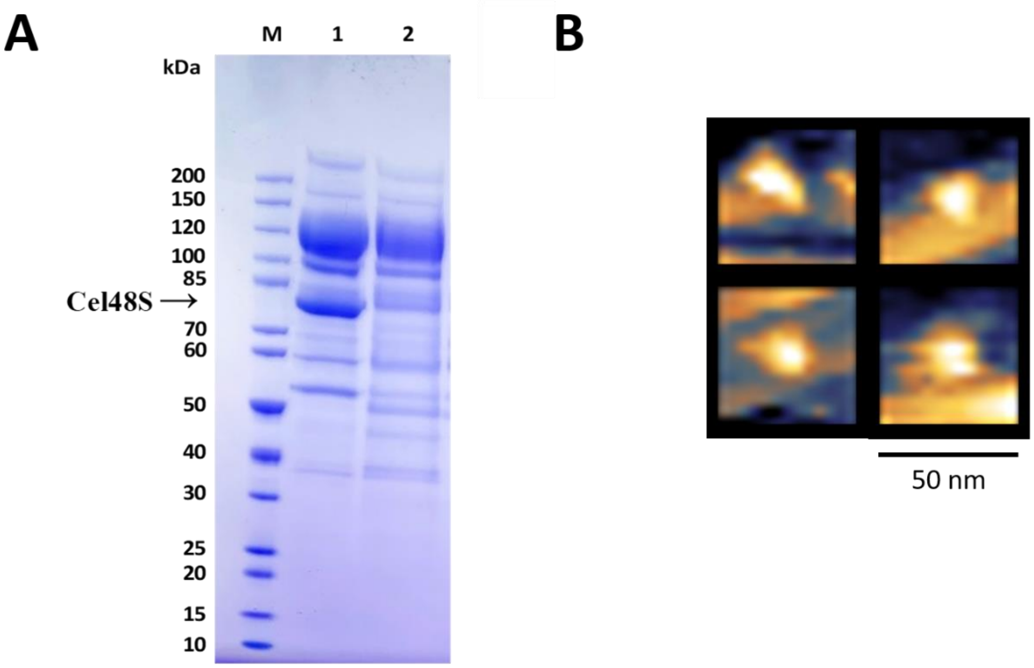

Fig. S8. SDS-PAGE analysis of purified wild type and mutant cellulosomes.

(A) Lane M indicates marker, lane 1 is wild type cellulosome and lane 2 is ΔCel48S. The Cel48S band is indicated by an arrow.

(B) Exemplary AFM height images of ΔCel48S cellulosomes.

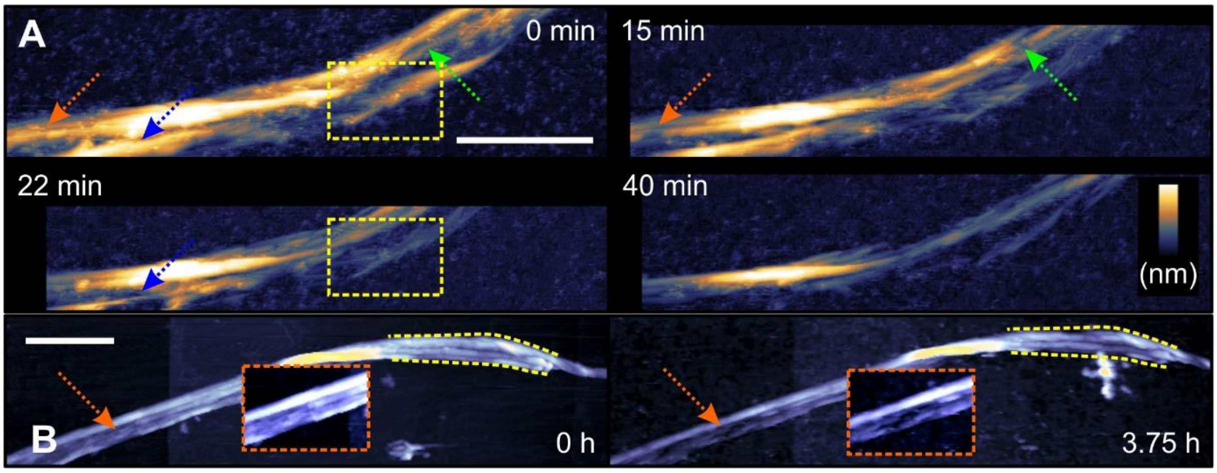

Fig. S9. AFM snapshots of cellulosomes degrading bacterial cellulose fibers.

(A) Exemplary areas of local fibril fragmentation are indicated by colored arrows. The color signifies the position before and after the fragmentation event. The yellow rectangle highlights an area where multiple fibrils are disentangled. Snapshots are extracted from Movie S1.

(B) ΔCel48S acting on bacterial cellulose. The degree of degradation is lower than that of the native system over a comparable time period but exhibits similar features, such as the disruption of cellulose fibers (marked with a yellow frame) and the excavation of cavities (highlighted in orange). Note that the contrast was increased by 20% for the zoomed inset to facilitate viewing. Scale bars are 250 nm. The false color scale used throughout is in panel A and height ranges are 22 nm, and 18 nm for panel A, and, B, respectively.

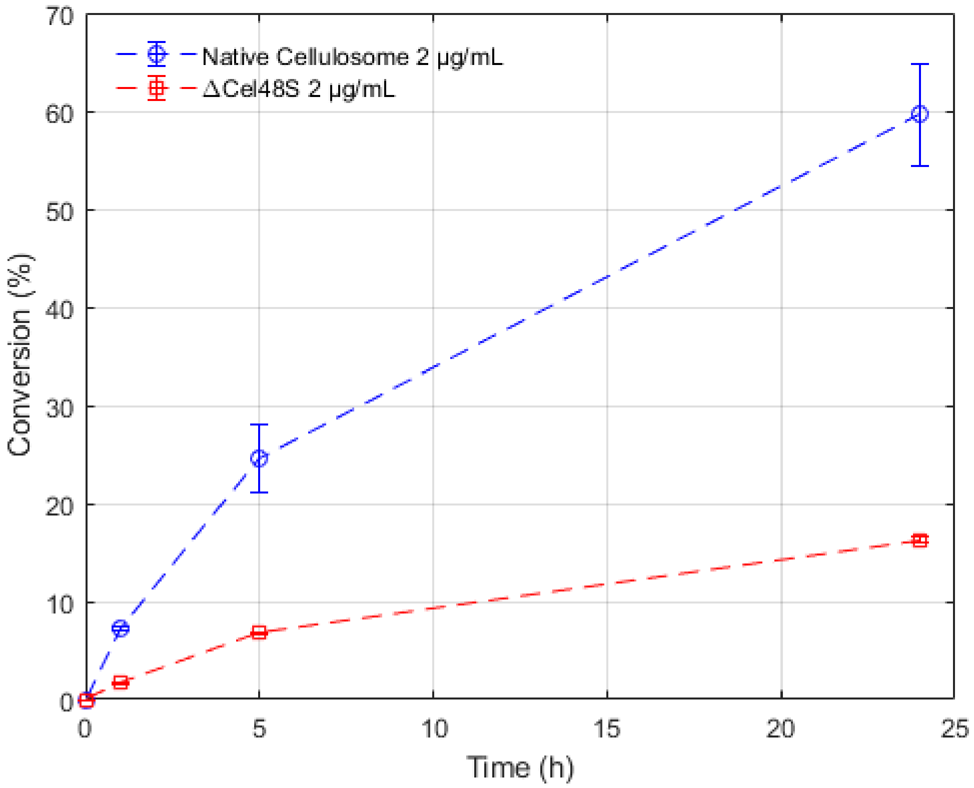

Fig. S10. Comparison of the conversion rates of native and mutant cellulosomes. Reactions were carried out using 1.0 mg/mL bacterial cellulose, and 2.0 μg/mL enzyme loadings at 55 °C, 500 rpm in cellulosome buffer. All the reactions were performed in duplicates.

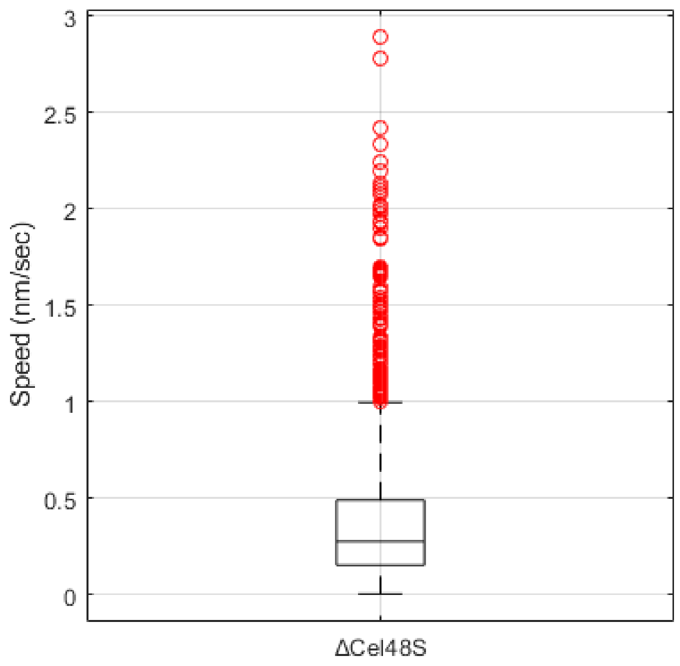

Fig. S11. Boxplot depicting the range of velocity observed for ΔCel48S cellulosomes. On average, the mutants center of mass moves at a velocity of 0.3 nm/s.

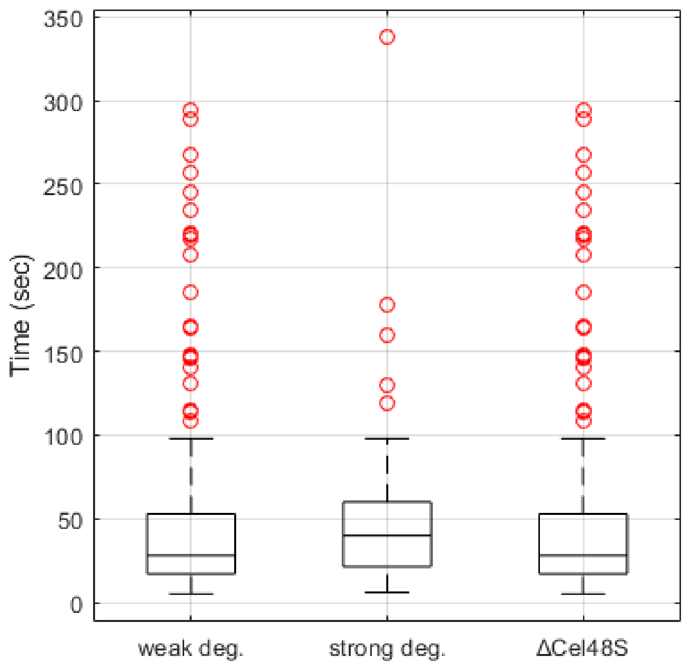

Fig. S12. Comparison of the typical residence times for cellulosomes engaged in different degradation processes.

On average, the S-type cellulosomes (middle) were found to be present on the fiber surface for 33 seconds, while both the W-type (left) and ΔCel48S cellulosomes (right) remained for 21 seconds.

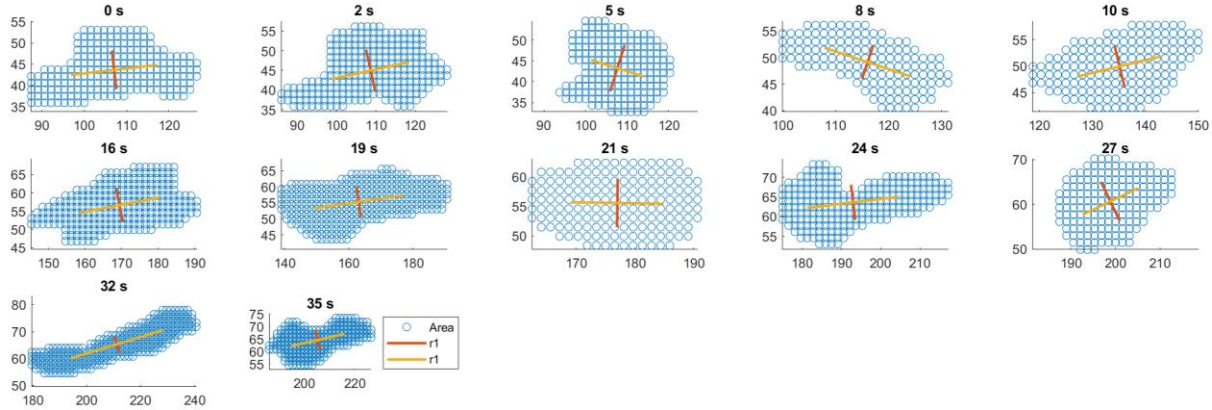

Fig. S13. Shape tracking example.

The sequence of a cellulosome being tracked for 35 sec in different frames, indicating its short ($\vec{r}_1$, red) and long axis ($\vec{r}_2$, orange) shows the time resolved shape changes.

Supporting Movie captions

Movie S1.

Real-time observations of the cellulosomes from *C. thermocellum* on bacterial cellulose. Cellulosomes are subdivided into moving/sitting molecules and are highlighted accordingly (green/red). Cellulosome was present at a concentration of about 2 µg/mL. Scale bar, time stamps and false color scale are included in the video.

Movie S2.

Prominent examples of massive volume degradation of fiber parts by the cellulosomes.

(a-f) Events of individual cellulosomes removing a substantial amount of material while either moving (a,c,e-f) or sitting (b,d) in dependency of the fiber surface roughness. Note that in (a) two distinct events can be observed starting at the 5:46:19 and 5:48:31 marks. The time resolution varied between 0.14 and 2 frames/sec. Scale bar, time stamps and false color scale are included in the video.

Movie S3.

Real-time observation of ΔCel48S cellulosomes acting on bacterial cellulose.

(a-c) Contrary to the native cellulosome (see Movie S1 for comparison), directional movement is absent from the population of ΔCel48S cellulosomes. Exemplary ΔCel48S molecules are highlighted in red. (d,e) Exemplary ΔCel48S cellulosomes analyzed using custom-developed MATLAB routine for shape, center of mass, and trajectory. ΔCel48S cellulosomes were present at a concentration of about 3 µg/mL. Image acquisition rate varied between 0.14 and 0.3 frames/sec. Scale bar, time stamps and false color scale are included in the video.

Movie S4.

Snapshots of real-time AFM observations of representative S-population cellulosomes.

(a-c) Cellulosome shapes exhibit periodic extension and compression during directional movement, resembling macromolecular crawl of protein with elastic transitions of conformation. The cellulosome responsible for the degradation is highlighted in green. (d-f) Highlighted cellulosomes (a-c) are analyzed for shape, center of mass, and trajectory using a custom-developed MATLAB routine. These cellulosomes (a, b, and d, e) are also featured in

Fig. 3C,D. Image acquisition rates varied between 0.14 and 0.3 frames/sec. The video includes scale bars, time stamps, and a false color scale for reference.

Supporting Dataset captions

Supporting Dataset 1

Exemplary GIFs showing the shape, center of mass and trajectory of several sitting cellulosomes.

Supporting Dataset 2

Exemplary GIFs showing the shape, center of mass and trajectory of several ΔCel48S cellulosomes.

Supporting Dataset 3

Exemplary GIFs showing the shape, center of mass and trajectory of several moving cellulosomes.

References

(1) Zajki-Zechmeister, K.; Kaira, G. S. S.; Eibinger, M.; Seelich, K.; Nidetzky, B. Processive Enzymes Kept on a Leash: How Cellulase Activity in Multienzyme Complexes Directs Nanoscale Deconstruction of Cellulose. *ACS Catal* **2021**, *11* (21), 13530–13542. https://doi.org/10.1021/acscatal.1c03465.

(2) Resch, M. G.; Donohoe, B. S.; Baker, J. O.; Decker, S. R.; Bayer, E. A.; Beckham, G. T.; Himmel, M. E. Fungal Cellulases and Complexed Cellulosomal Enzymes Exhibit Synergistic Mechanisms in Cellulose Deconstruction. *Energy Environ Sci* **2013**, *6* (6), 1858. https://doi.org/10.1039/c3ee00019b.

(3) Bayer, E. A.; Lamed, R. Ultrastructure of the Cell Surface Cellulosome of *Clostridium Thermocellum* and Its Interaction with Cellulose. *J Bacteriol* **1986**, *167* (3), 828–836.

(4) Eibinger, M.; Ganner, T.; Plank, H.; Nidetzky, B. A Biological Nanomachine at Work: Watching the Cellulosome Degrade Crystalline Cellulose. *ACS Cent Sci* **2020**, *6* (5), 739–746. https://doi.org/10.1021/acscentsci.0c00050.

(5) Zajki-Zechmeister, K.; Eibinger, M.; Nidetzky, B. Enzyme Synergy in Transient Clusters of Endo- and Exocellulase Enables a Multilayer Mode of Processive Depolymerization of Cellulose. *ACS Catal* **2022**, *12* (17), 10984–10994. https://doi.org/10.1021/acscatal.2c02377.

(6) Amyot, R.; Flechsig, H. BioAFMviewer: An Interactive Interface for Simulated AFM Scanning of Biomolecular Structures and Dynamics. *PLoS Comput Biol* **2020**, *16* (11), e1008444. https://doi.org/10.1371/journal.pcbi.1008444.
