# Supplementary figures and images for: Mechanochemical Coupling of Catalysis and Motion in a Cellulose-Degrading Multienzyme Nanomachine
Source: ACS Catal. 2024 Feb 6;14(4):2656–63. doi: 10.1021/acscatal.3c05653 (PMC10877591; doi:10.1021/acscatal.3c05653)

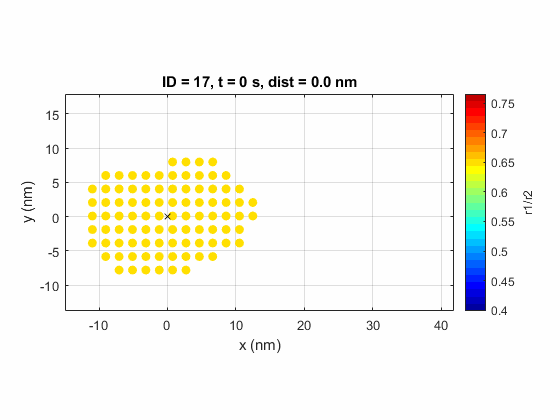

Supplement: Supplementary file 6 — cs3c05653_si_006.zip [file cs3c05653_si_006.zip › Supporting_Dataset_1/17.gif]

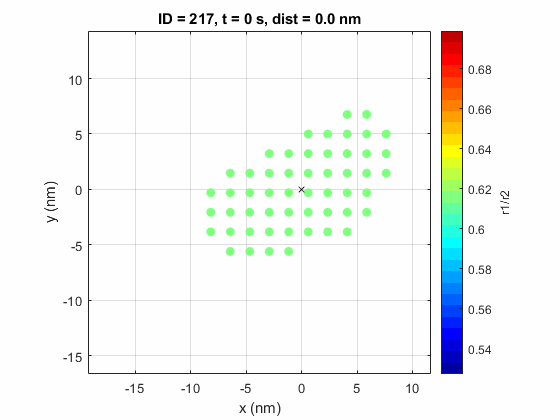

Supplement: Supplementary file 6 — cs3c05653_si_006.zip [file cs3c05653_si_006.zip › Supporting_Dataset_1/217.gif]

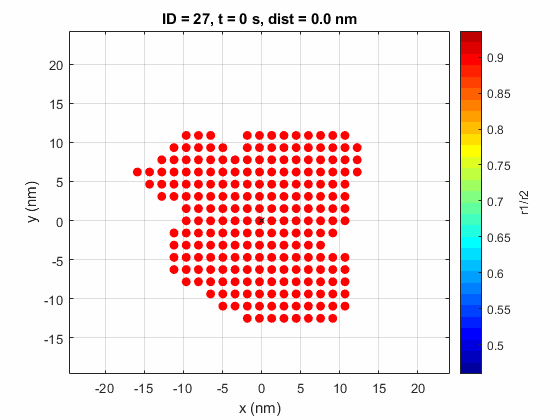

Supplement: Supplementary file 6 — cs3c05653_si_006.zip [file cs3c05653_si_006.zip › Supporting_Dataset_1/27.gif]

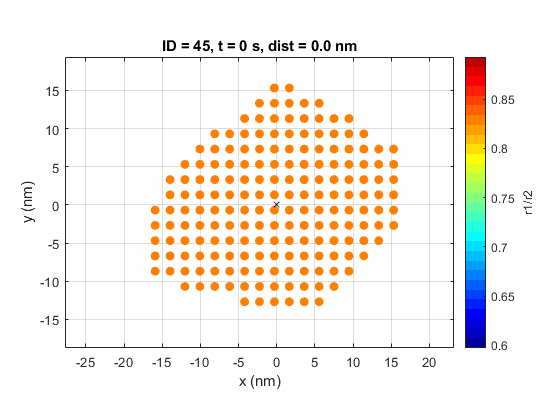

Supplement: Supplementary file 6 — cs3c05653_si_006.zip [file cs3c05653_si_006.zip › Supporting_Dataset_1/45.gif]

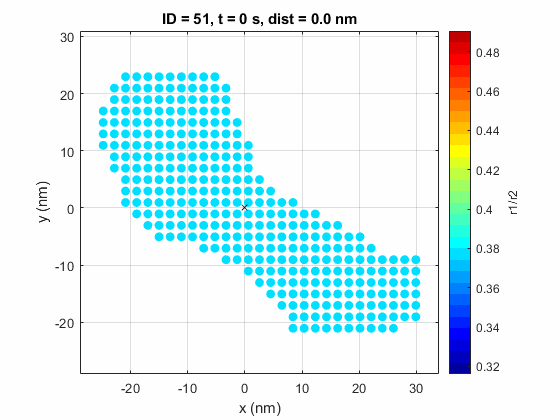

Supplement: Supplementary file 6 — cs3c05653_si_006.zip [file cs3c05653_si_006.zip › Supporting_Dataset_1/51.gif]

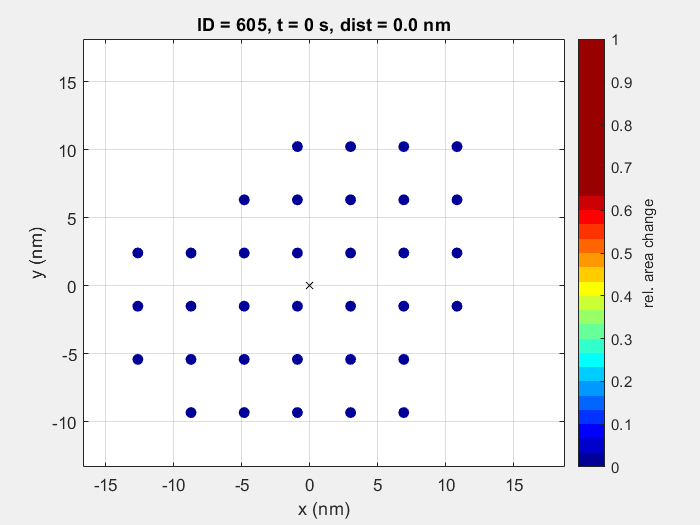

Supplement: Supplementary file 7 — cs3c05653_si_007.zip [file cs3c05653_si_007.zip › Supporting_Dataset_2/605.gif]

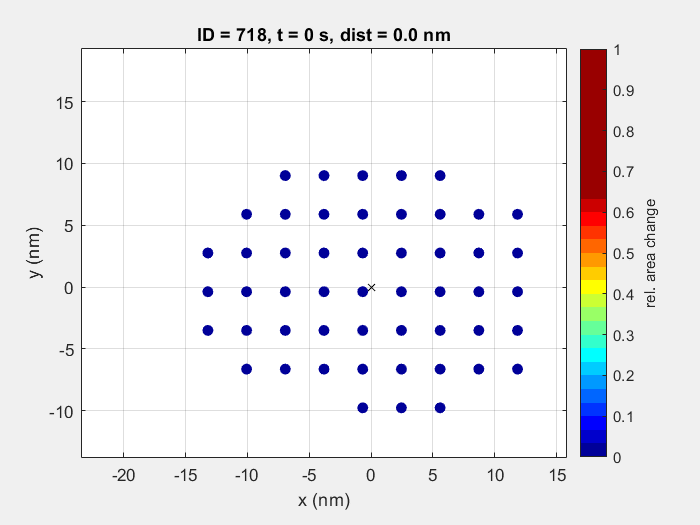

Supplement: Supplementary file 7 — cs3c05653_si_007.zip [file cs3c05653_si_007.zip › Supporting_Dataset_2/718.gif]

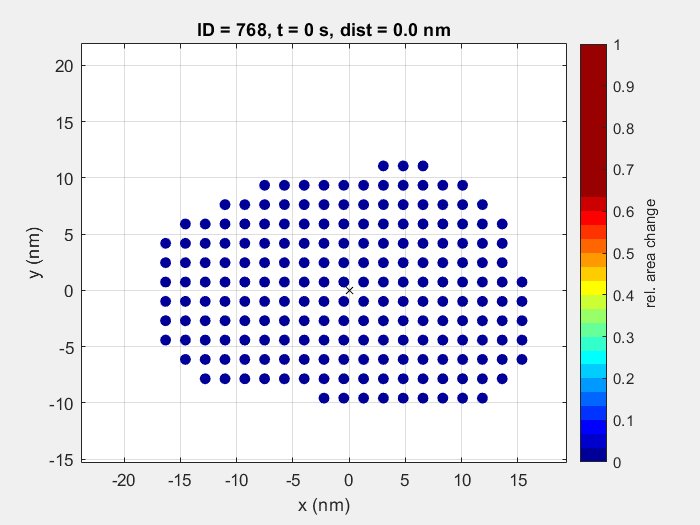

Supplement: Supplementary file 7 — cs3c05653_si_007.zip [file cs3c05653_si_007.zip › Supporting_Dataset_2/768.gif]

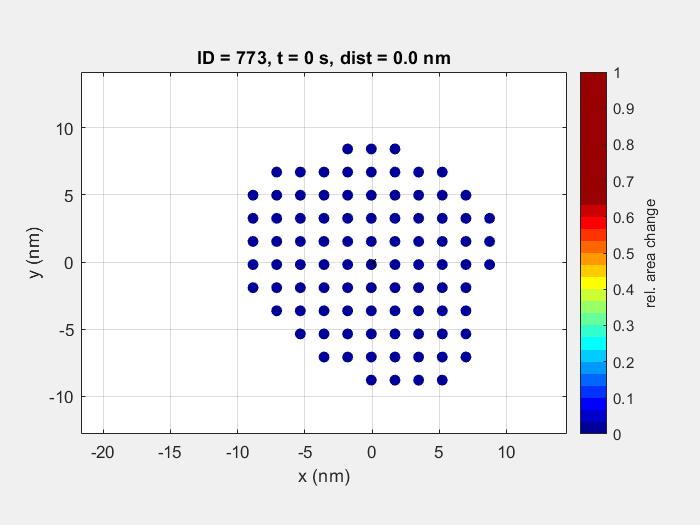

Supplement: Supplementary file 7 — cs3c05653_si_007.zip [file cs3c05653_si_007.zip › Supporting_Dataset_2/773.gif]

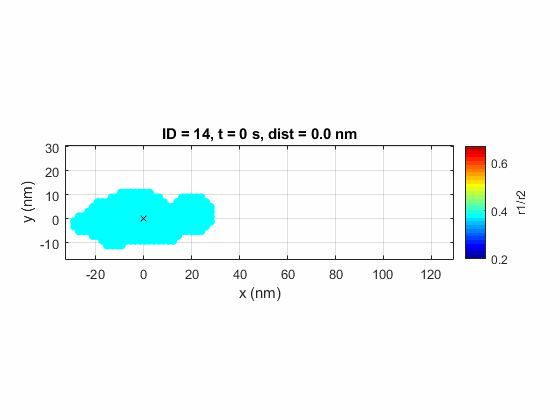

Supplement: Supplementary file 8 — cs3c05653_si_008.zip [file cs3c05653_si_008.zip › Supporting_Dataset_3/14.gif]

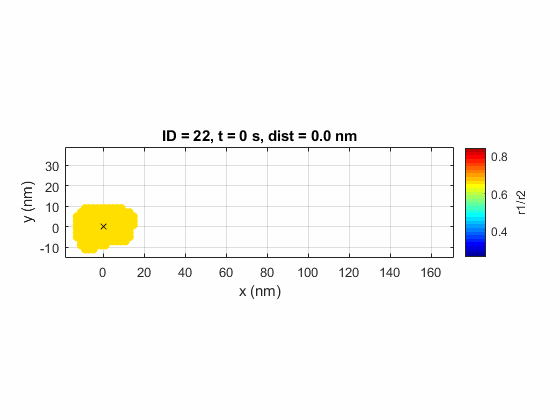

Supplement: Supplementary file 8 — cs3c05653_si_008.zip [file cs3c05653_si_008.zip › Supporting_Dataset_3/22.gif]

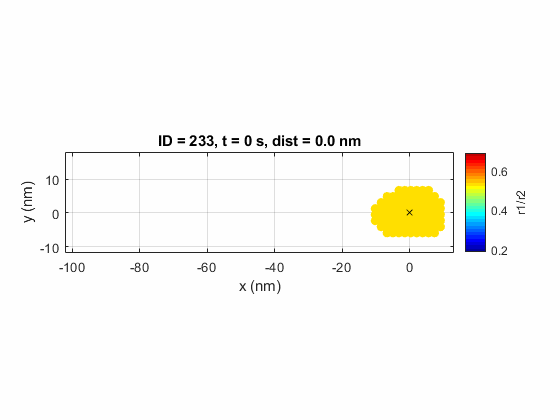

Supplement: Supplementary file 8 — cs3c05653_si_008.zip [file cs3c05653_si_008.zip › Supporting_Dataset_3/233.gif]

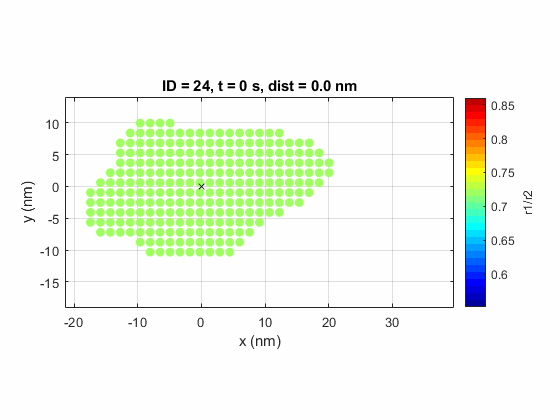

Supplement: Supplementary file 8 — cs3c05653_si_008.zip [file cs3c05653_si_008.zip › Supporting_Dataset_3/24.gif]

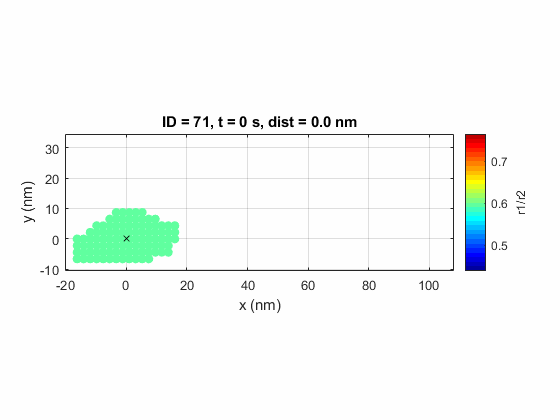

Supplement: Supplementary file 8 — cs3c05653_si_008.zip [file cs3c05653_si_008.zip › Supporting_Dataset_3/71.gif]
